# Local Flexibility of a New Single-Ring Chaperonin Encoded by Bacteriophage AR9 *Bacillus subtilis*

**DOI:** 10.3390/biomedicines10102347

**Published:** 2022-09-21

**Authors:** Olga S. Sokolova, Evgeny B. Pichkur, Ekaterina S. Maslova, Lidia P. Kurochkina, Pavel I. Semenyuk, Petr V. Konarev, Valeriya R. Samygina, Tatiana B. Stanishneva-Konovalova

**Affiliations:** 1 Faculty of Biology, MSU-BIT Shenzhen University, Shenzhen 518172, China; 2 Complex of NBICS Technologies, National Research Center “Kurchatov Institute”, 123098 Moscow, Russia; 3 Faculty of Biology, Lomonosov Moscow State University, 119991 Moscow, Russia; 4 Belozersky Institute of Physico-Chemical Biology, Lomonosov Moscow State University, 119234 Moscow, Russia; 5 Shubnikov Institute of Crystallography of FSRC “Crystallography and Photonics”, RAS, 119333 Moscow, Russia

**Keywords:** molecular chaperones, chaperonins, Cryo-EM, bacteriophages

## Abstract

Chaperonins, a family of molecular chaperones, assist protein folding in all domains of life. They are classified into two groups: bacterial variants and those present in endosymbiotic organelles of eukaryotes belong to group I, while group II includes chaperonins from the cytosol of archaea and eukaryotes. Recently, chaperonins of a prospective new group were discovered in giant bacteriophages; however, structures have been determined for only two of them. Here, using cryo-EM, we resolved a structure of a new chaperonin encoded by gene 228 of phage AR9 *B. subtilis*. This structure has similarities and differences with members of both groups, as well as with other known phage chaperonins, which further proves their diversity.

## 1. Introduction

Chaperonins are protein complexes that promote the proper folding of newly synthesized proteins and prevent the aggregation of denatured proteins in an ATP-dependent manner. Until recently, two major groups of chaperonins were distinguished. Group I chaperonins, found in the cytoplasm of bacteria and in endosymbiotic organelles of eukaryotes, are homo-oligomeric complexes and require co-chaperonins to function [1]. Group II chaperonins, found in the cytoplasm of archaea and eukaryotes, are hetero-oligomers and function without a co-chaperonin [2,3]. Representatives of both groups have, as a rule, a double-ring structure with a cavity for protein folding in each ring. Recently, chaperonins of the prospective new group were discovered in giant bacteriophages [4,5,6,7,8]. Some bacteriophages are known to use a host cell chaperonin to fold their own capsid proteins, with phage λ using the entire GroEL-GroES complex, while T4 and RB49 encode their own GroES orthologs, gp31 and CocO, respectively [9,10,11]. This led investigators to the idea that perhaps viruses may also have their own protein folding apparatus. The first gene encoding a GroEL ortholog was found in the genome of a giant bacteriophage EL *Pseudomonas aeruginosa*. The protein is encoded by gene 146 and its product has only 25% homology to the amino acid sequence of GroEL, which is quite low compared to 80% identity within group I chaperonins [4]. Gene 146 was successfully cloned and expressed in *E. coli* cells. Purified recombinant protein was found to consist of 14 identical gp146 subunits arranged in two heptameric rings, i.e., its architecture is similar to the GroEL tetradecamer. All known chaperonins, including phage chaperonins, are conservative proteins, subunits of which are composed of three domains: equatorial, intermediate and apical. EL chaperonin possesses a functional activity towards endolysin (a protein used by phages to disrupt the peptidoglycan of the bacterial cell wall and exit from the cell). As shown in vitro, it is able to prevent the thermal aggregation of endolysin both in the presence and absence of ATP. Unlike GroEL, EL chaperonin does not require a co-chaperonin to function [12].

A study of another putative chaperonin of bacteriophage OBP *Pseudomonas fluorescens* (gp246) revealed that, according to the phylogenetic tree, it is also an ATP-dependent GroEL-like protein that functions without a co-chaperonin, but has a number of unique features [13], including the fact that it only forms a single-ring barrel. Three-dimensional reconstruction obtained by cryo-electron microscopy and single particle analysis demonstrated that its structure consists of seven subunits. The equatorial domains of OBP chaperonin possess a clear C7 symmetry and closely resemble the overall structure of the one ring of GroEL [14]. At the level of intermediate domains, which connect equatorial and apical domains, the C7 symmetry diminishes, resulting in the appearance of an asymmetrical pattern: three subunit pairs (A–B) and one unpaired subunit (C), at the level of apical domains [15]. Subunit A resembles the T-state (low affinity to nucleotides) of GroEL, while subunit B is close to the Rs1 (ATP-bound) state of GroEL. The C subunit was poorly resolved, indicating its highly dynamic nature and continuous transition between A and B states. A similar asymmetric subunit arrangement was later observed for the apical domains of the double-ring EL chaperonin [16]. In addition, it was demonstrated that in the presence of ATP and at physiological salt concentrations, EL chaperonin dissociates into two separate rings [16]. According to the proposed hypothetical mechanism, next, ATP hydrolysis occurs, which allows the folded protein to be released. After the dissociation of ADP, chaperonin again re-establishes a double-ring conformation [16].

The third GroEL-like chaperonin was recently predicted based on the genome of phage AR9 *B. subtilis* [17]. The putative recombinant chaperonin (gp228) has been obtained and purified, and its low-resolution structure revealed that it is a single-ring heptamer. Like EL and OBP chaperonins, the new AR9 chaperonin does not require a co-chaperonin to function and provides a refolding of the denatured substrate protein, endolysin, in an ATP-dependent manner. It is important to understand how single-ring chaperonins without co-chaperonins could prevent the aggregation of substrates as well as fold them.

Here, we used the cryo-EM approach and molecular modeling to solve the high-resolution structure of AR9 chaperonin in the apo-state. The structure of the new viral chaperonin has been compared to the structures of other viral chaperonins and further proves the diversity of this important class of proteins.

## 2. Materials and Methods

### 2.1. Protein Purification

The gp228 was expressed in *E. coli* cells, as described elsewhere [17]. The recombinant bacteria were grown in LB medium at 37 °C to an optical density of 0.6 at 600 nm, induced with 1 mM of isopropyl-β-D-thiogalactopyranoside (IPTG), and additionally incubated at 25 °C for 3.5 h. Cell pellet was re-suspended in 50 mM Tris-HCl buffer (pH 7.5) and sonicated for 2–3 min, followed by centrifugation. Nucleic acids were pelleted from supernatant with 3% (wt/vol) streptomycin sulfate at 4 °C for 1 h and centrifuged. The most part of the cellular proteins were precipitated from the supernatant with 40% (wt/vol) ammonium sulfate and removed by centrifugation. Recombinant gp228 was precipitated from the supernatant by increasing the concentration of ammonium sulfate to 50%, and fractionated on a Q-Sepharose column (Cytiva Sweden AB, Uppsala, Sweden) by a linear gradient from 100 to 500 mM NaCl in 50 mM Tris-HCl buffer (pH 7.5). The eluate fractions containing pure gp228 were pooled and concentrated using an Amicon 100 ultrafiltration device (molecular weight cutoff (MWCO) 100,000 Da, Millipore, Burlington, MA, USA), and transferred into 50 mM Tris-HCl buffer (pH 7.5), 10 mM MgCl_2_, 100 mM KCl [17].

### 2.2. Cryo-EM Data Acquisition

Purified protein (3 μL) was applied to glow-discharged Quantifoil R1.2/1.3 grids (Quantifoil, Großlöbichau, Germany) and plunge-frozen in a Vitrobot Mark IV (Thermo Fisher Scientific, Waltham, MA, USA) at 4.5 °C. Images were taken on a Titan Krios cryo-electron microscope (Thermo Fisher Scientific, USA) equipped with a Falcon II direct electron detector (Thermo Fisher Scientific, USA) with an accelerating voltage of 300 kV. A total of 3184 stacks of 20 images each were acquired. The size of the images was 4096 × 4096 pixels at a resolution of 1.107 Å/pix. The electron dose absorbed by each image in the stack was 4 electrons per Å^2^.

### 2.3. Image Processing

Data processing was carried out using the RELION-3.0.8 program [18]. Using the MotionCor2 program [19], the anisotropic drift of the sample caused by the action of an electron beam was corrected. The first and last image in each stack were excluded from further processing, as they contain artifacts caused by the operation of electron microscope shutters. A total of 3184 two-dimensional images were obtained, for which the signal intensity distribution was taken into account depending on the electron dose absorbed by the sample [20]. CTF parameters were evaluated using the Gctf program [21]. The set of images was processed in the SPHIRE-crYOLO program [22]. A total of 1,106,416 coordinates were selected and subjected to 2D classification, after which 689,058 coordinates were selected for further processing. The second stage of projection selection was carried out using three-dimensional (3D) classification into 5 different classes. The most populated class containing 145,972 particles was refined with and without C7-symmetry imposed. The estimated resolution was 4.49 Å for the symmetry-free model and 3.99 Å for the C7-symmetrical structure.

To further improve the resolution, the motion of individual protein particles in the sample was corrected for the original dataset using the available reconstruction as a reference. Together with CTF-refinement, this allowed us to reach a 3.77 Å resolution for the C7-symmetrical model.

To study the conformational mobility of individual subunits, we used the symmetry expansion procedure implemented in RELION. Two masks were created: around the entire heptamer and around a single subunit. Subtraction of a mask for a single subunit from the heptamer mask allowed to ensure a mask for 6 subunits. Using the “relion_particle_symmetry_expand” command, we obtained 7 times more images of aligned subunits. Various projection images from the modified map with only 6 subunits were generated according to the particle orientation images and subtracted from the corresponding 2D images with extended symmetry. As a result, images containing the signal of only one subunit were obtained. These images were subjected to 3D classification in RELION. The atomic model of a single subunit was fit into these classes using the UCSF ChimeraX program and the specialized ISOLDE tool [23].

### 2.4. Molecular Modeling

On the basis of the map, an atomistic model of the AR9 chaperonin heptamer was reconstructed. Homology modeling of the monomer using a GroEL structure as a template was performed in the Swiss-model [24], then, the structure of the monomer was flexibly fitted into the density map using Phenix [25].

### 2.5. Small-Angle X-ray Scattering

Small-angle X-ray scattering (SAXS) measurements were performed at the P12 beamline of the European Molecular Biology Laboratory (EMBL) at the PETRA III storage ring, DESY Hamburg [26]. The gp228 sample at the concentration range of 0.5 to 7.0 mg/mL was tested. The solutions were loaded using a robotic sample changer [27] into a flow-through capillary of 1.7 mm diameter. The data were recorded using a Pilatus 6M detector (DECTRIS, Baden-Daettwil, Switzerland) with 20 × 0.05 s exposure time at the sample, to a detector distance of 3.10 m and a wavelength of 0.124 nm covering the momentum transfer range from 0.02 < s < 7.0 nm^−1^. The temperature was kept at 20 °C. The data collection and reduction were performed using the BECQUEREL [28] and SASFLOW pipeline [29], including the comparison of frames for radiation damage, averaging, and buffer subtraction. The averaged frames were normalized to the transmitted beam using a beamstop with an integrated photodiode [30]. No measurable radiation damage was detected by the comparison of successive time frames.

The radius of gyration (R_g_), maximum size of the particle (D_max_) and the pair distribution function p(r) were evaluated using the program GNOM [31]. The excluded volume of hydrated protein molecule (V_p_) was estimated within the Porod approximation for homogeneous particles. A low-resolution ab initio bead model of the gp228 protein was constructed using the program DAMMIN [32], where the simulated annealing (SA) minimization algorithm was used to build a compact interconnected configuration of beads with the best fit to the experimental data.

The theoretical scattering intensities from the refined cryo-EM models with C1 and C7 symmetries were calculated using the CRYSOL program [33] and compared to the experimental data. The flexibility of apical domains in the cryo-EM model with C7 symmetry was assessed using the program EOM [34].

## 3. Results

### 3.1. Apo-Form of AR9 Chaperonin Has a Flexible 3D Structure

**Figure 1:**
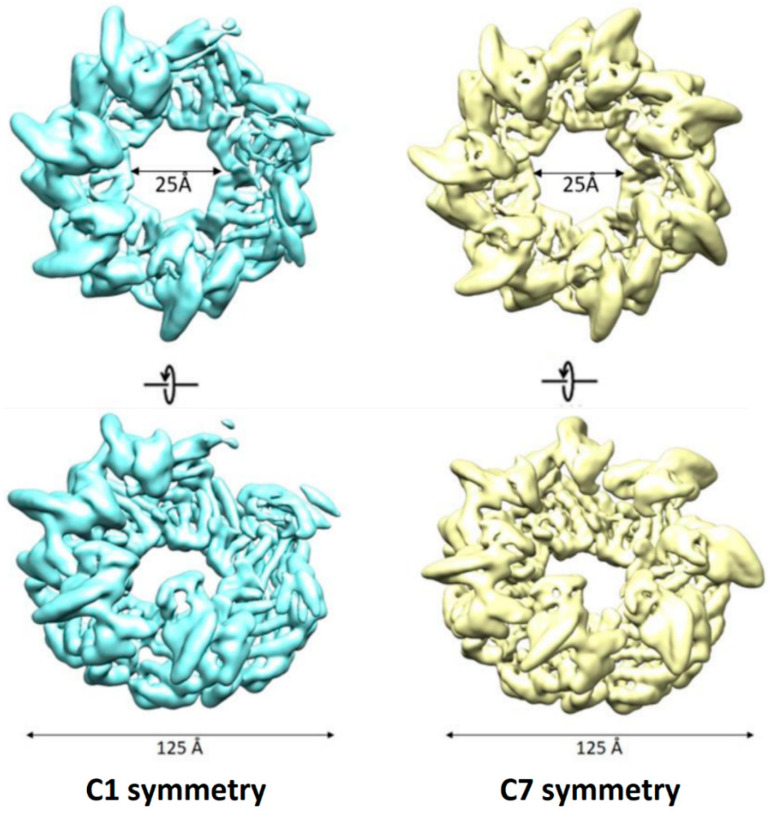
Cryo-EM density maps of AR9 chaperonin with no symmetry applied (**left**) and with C7 symmetry (**right**).

For AR9 chaperonin consisting of seven gp228 subunits, we obtained two cryo-EM maps: with no symmetry imposed (resolution 4.49 Å) and with C7 symmetry (resolution 3.99 Å) (Figure 1).

**Figure 2:**
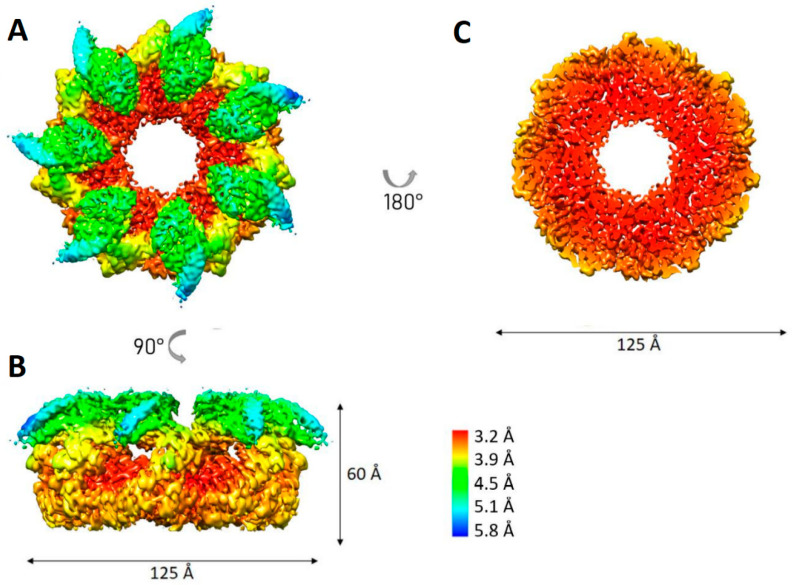
Local resolution estimation for C7 symmetric density map of AR9 chaperonin. (**A**) Top view; (**B**) side view; (**C**) slice through the equatorial domains.

The symmetry-free map demonstrated a loss of scattering density in the region of three adjacent apical domains. As in the case of the OBP chaperonin, the region poorly resolved in the 3D reconstruction may indicate its high conformational dynamics [15]. However, despite these conformational dynamics, and unlike the OBP chaperonin, in the C7-symmetrical structure, all subunits are distinguishable, so we used it for further refinement. Correction of particle motion in the sample and additional refinement of the CTF parameters for each particle made it possible to refine the C7-symmetric structure to a resolution of 3.77 Å (Figure 2). The local resolution in the region of the equatorial domains reaches 3.2 Å, while in the apical domains, the resolution drops to 5.8 Å. There are also some differences in the resolution score for three of the seven apical domains, which is consistent with the observed density loss of the three apical domains in the map without symmetry.

### 3.2. Full-Atom Model of the Apo-Form of the AR9 Chaperonin Demonstrates More Stability than That of the OBP Chaperonin

**Figure 3:**
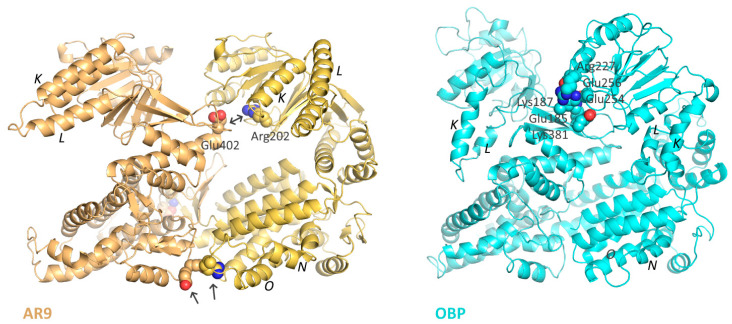
Inter-subunit contacts in the apo-form of the AR9 chaperonin (**left**) and the OBP chaperonin (**right**). Arrows show important inter-subunit salt bridges; the helices K, L, N, O and the key Arg202-Glu402 pair are labeled.

Based on the obtained reconstruction of the AR9 chaperonin, a full-atom model of the heptamer was constructed. For this purpose, homologous modeling of a monomer was performed using the structure of the bacterial GroEL as a template; the resulting structure was corrected and fitted into the density map of the apo-form using the PHENIX package and flexible fitting. This allowed us to demonstrate the unusual position of the K and L helices. However, unlike the other heptameric phage chaperonin OBP, the R197-E386 ion pair key for GroEL functioning [35] is retained in the chaperonin AR9 structure (R202-E402 in AR9 chaperonin) (Figure 3). In addition, the formation of additional (compared to GroEL and other phage chaperonins) interactions between the equatorial domains of neighboring subunits near the N and O helices is possible, which can further stabilize the chaperonin.

### 3.3. Conformational Variability among the Subunits

**Figure 4:**
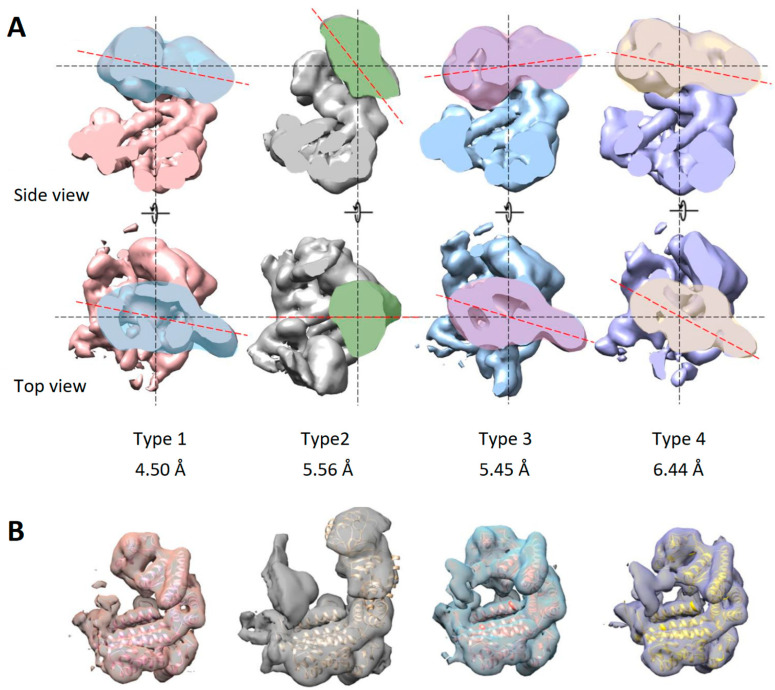
Conformational variability the apo-form of the AR9 chaperonin. (**A**) Subunit conformations resolved at 4.50 Å, 5.56 Å, 5.45 Å and 6.44 Å. The red dotted line indicates the orientation of the apical domain. (**B**) Fitting of atomic models into density maps of identified subunit conformations.

To further study the conformational mobility of subunits, one subunit was isolated by masking and used as a reference for three-dimensional classification. Previously, the set of particles was expanded using the “symmetry expansion” function in the RELION program. After three-dimensional classification, 15 classes were obtained, of which 13 displayed an expected three-domain architecture of a single subunit. Some of the classes presented a very similar subunit conformation. The particles from such classes were grouped together and used for further refinement of a particular conformation. As a result, four subunit conformations were identified at resolutions 4.50 Å, 5.56 Å, 5.45 Å and 6.44 Å (Figure 4A).

The first conformation corresponds to a subunit in the average C7-symmetrical model. Relative to the average position, the greatest changes in other conformations occur in the apical domains. Subunits of the second conformation have an elevated apical domain; GroEL subunits adopt a similar conformation when bound to GroES. The third type included subunits whose apical domain was lowered to the center of mass of the chaperonin ring. The fourth type was formed by subunits with the apical domain rotated around the axis of the subunit to the right. Next, for each conformation, the atomic model fitting was performed (Figure 4B). The mobility of the equatorial domain is limited by inter-subunit contacts, while the apical domains play a major role in the mobility of the apo-form of chaperonin.

**Figure 5:**
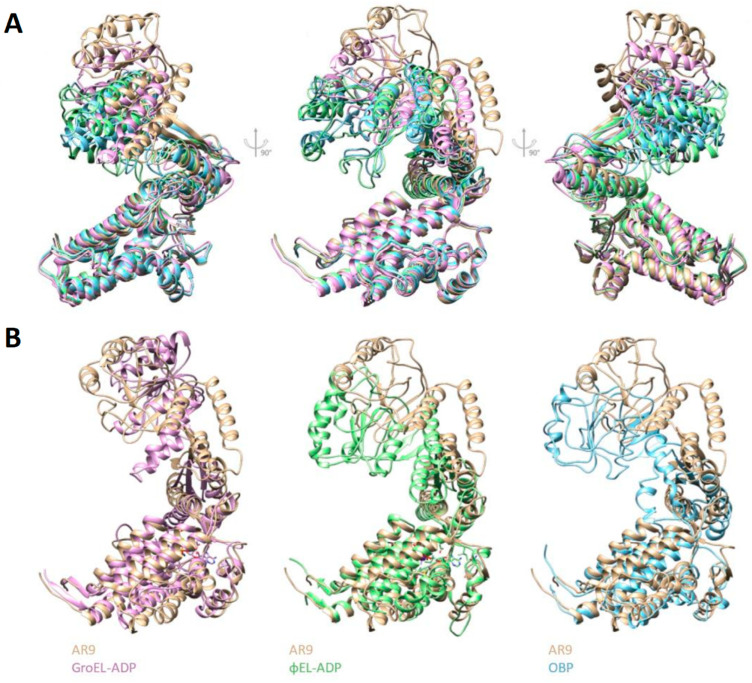
Comparison of subunit conformations of phage chaperonins and GroEL. (**A**) Alignment of 4 subunit conformations of the AR9 chaperonin. The first to the fourth types are colored blue, tan, green and pink, respectively. (**B**) Comparison of the second type subunit with GroEL and other phage chaperonins.

Figure 5A shows the alignment of four conformations and the main differences between them. Similar movements of the apical domain are present in the cycle of the bacterial chaperonin, GroEL, upon its binding to GroES. However, the second type subunit (with the most elevated position of the apical domain) does not repeat any of the putatively similar conformational states of known bacteriophage chaperonins or of the bacterial chaperonin, GroEL (Figure 5B).

### 3.4. Apo-AR9 Cryo-EM Structure Agrees with Solution Scattering Data

**Figure 6:**
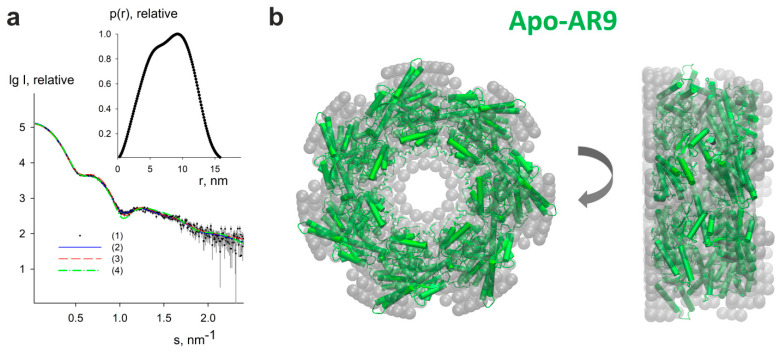
SAXS study of Apo-AR9. (**a**) Small-angle X-ray scattering patterns from Apo-AR9 chaperonin. Curve (1)—experimental data are displayed as dots with error bars, curve (2)—the fit from ab initio model obtained by DAMMIN [32] is shown as a blue solid line, curves (3) and (4)—theoretical scattering curves calculated by CRYSOL [33] from cryo-EM models with C1 and C7 symmetry are shown as red dashed and green dashed-dotted lines, respectively. The plots display the logarithm of the scattering intensity as a function of the momentum transfer. The inset displays the distance distribution function estimated by GNOM [31]. (**b**) Ab initio bead model of Apo-AR9 chaperonin obtained by DAMMIN (gray semitransparent spheres) overlapped with the heptameric cryo-EM model in the cartoon representation (green color). The right view was obtained by a 90° clockwise rotation around the vertical axis. The figure was generated using the VMD program [37].

Information about apo-form of AR9 chaperonin in solution has been obtained from SAXS experiments. The processed scattering data and the computed distance distribution function are displayed in Figure 6a. The excluded volume V_p_ of the particle (850 ± 50) nm^3^ suggests its predominantly heptameric state, which is in agreement with an empirical finding for globular proteins that the hydrated volume in nm^3^ should numerically be about twice larger than the molecular mass in kDa [36] (the theoretical molecular mass of the monomer is 61.4 kDa). The experimental R_g_ and D_max_ (6.0 ± 0.2 nm and 16.5 ± 0.5 nm, respectively) point to a rather compact structure. The distance distribution function p(r) for Apo-AR9 (Figure 6a, inset) is also consistent with the compact shape of the protein. The rather asymmetric view of p(r) function with the shift of its peak towards larger distances is typical for particles with a cavity in the central part of the structure.

The macromolecular shape of the Apo-AR9 chaperonin has been reconstructed by ab initio modeling using only the experimental X-ray scattering data.

An ab initio low resolution model displayed in Figure 6b has been generated by DAMMIN (see Methods for details). A typical low-resolution shape of Apo-AR9 reconstructed ab initio (Figure 6b) nicely fits the experimental data with the discrepancy χ^2^ = 1.05 (Figure 6a, curve 2, solid blue line).

The scattering intensities calculated from the refined cryo-EM models with C1 and C7 symmetries are displayed in Figure 6a, curves (3) and (4) shown with red dashed and green dashed-dotted lines, respectively. While the cryo-EM model without symmetry provides a poor fit to the data with χ^2^ = 20.04, the symmetric model fits the data reasonably well with χ^2^ = 1.35. The small deviations from the data at s values around 1.0 nm^−1^ can be explained by a moderate flexibility of the apical domains. Indeed, the ensemble optimization method (EOM) [34] allowed us to slightly improve the fit quality (χ^2^ = 1.29) using the randomized pool of symmetric models, where the equatorial and intermediate domains were fixed as in the cryo-EM model, and the apical domains (residues 189-387) were rotated while keeping the contacts between the domains. EOM found two conformer populations, a compact one with Rg around 5.9 nm (accounting for 60% of the total population) and a more extended one with Rg close to 6.1 nm (with 40% of the total population). At the same time, the symmetric cryo-EM model overlaps well with the ab initio shape (Figure 6b). Thus, we confirm that our symmetric cryo-EM structure is consistent with the solution scattering data.

## 4. Discussion

The AR9 chaperonin is the third viral chaperonin discovered and described by our scientific groups [15,17,38]. It is a homooligomer composed of seven gp228 subunits, which makes it similar to a single ring of the group I chaperonins. Single-ring chaperonins are less common in nature; however, single-ring forms of the mutant type I chaperonin GroEL [39,40,41] and mitochondrial chaperonin [42] are known to have functional activity. Moreover, it has been recently established that in the ATPase cycle of the bacterial GroEL-GroES system, there is a temporary separation of the chaperonin rings [43], and in the mitochondrial chaperonin reaction cycle, mHsp60-mHsp10, both single- and double-ring forms function simultaneously [44].

The main function of any chaperonin is to provide its inner cavity to the substrate. It is ensured by group I and II chaperonins through ATP-dependent transitions between open and closed conformations. In contrast, phage chaperonins are always in the open conformation. Yet they are functional and can suppress the thermal aggregation of phage endolysins and to fold denatured proteins regenerating their enzymatic activity in an ATP-dependent manner [17,38]. In addition, two types of phage chaperonins, the double-ring EL and the single-ring OBP, were demonstrated to stimulate α-synuclein amyloid transformation in an ATP-dependent manner and, on the contrary, completely prevent α-synuclein fibrillization in the absence of ATP [45].

The best resolution in our 3D model of AR9 chaperonin was achieved in the equatorial domains (3.2 Å), since their mobility is limited. The α-helices, which predominantly form the equatorial domains of the chaperonin ring similar to those of GroEL, are clearly visible. Increasing the mobility of the intermediate and apical domains due to conformational rearrangements and to increasing degrees of freedom, results in a lower resolution (4.5–5 Å). The classification of masked subunits allowed us to identify four subunit conformations in the apo-form of the AR9 chaperonin, indicating the conformational flexibility of this single-ring variant. This is reminiscent of the results obtained for GroEL, where a single mutation disrupted the intra-ring symmetry, resulting in the conversion of the allosteric switch of GroEL from concerted to sequential [46].

Comparison of the obtained reconstruction with previously published reconstructions of OBP [15] and EL chaperonins [16] demonstrated a difference in the subunit arrangement. While the subunits of OBP and EL chaperonins are arranged in three pairs with one unpaired subunit, such a pattern is not observed for the chaperonin under study. The similar structure of the equatorial chaperonin domains confirms the data on their conservation.

To confirm our cryo-EM model, we employed SAXS as a widely used method for integrative structural studies of large macromolecular complexes, including different chaperonins [47,48,49,50]. This structural approach provides direct information on the size, folding state, and flexibility of biomolecules at quasi-native conditions at room temperature [51].

Complementary synchrotron SAXS data from gp228 are in good agreement with the cryo-EM reconstruction. SAXS modeling reveals C7 symmetry of the protein in the native state. This finding confirms that Apo-AR9 has distinct features from OBP chaperonin belonging to the same group of phage chaperonins. Further research will help to understand how the diverse structural features of members of this new group affect their functional cycle.

## Data Availability

Not applicable.
